# Mesenchymal Stem Cells as a Bio Organ for Treatment of Female Infertility

**DOI:** 10.3390/cells9102253

**Published:** 2020-10-08

**Authors:** Sahar Esfandyari, Rishi Man Chugh, Hang-soo Park, Elie Hobeika, Mara Ulin, Ayman Al-Hendy

**Affiliations:** 1 Department of Surgery, University of Illinois at Chicago, 820 South Wood Street, Chicago, IL 60612, USA; sesfan2@uic.edu (S.E.); rmchugh@uic.edu (R.M.C.); hspark@uic.edu (H.-s.P.); mulin2@uic.edu (M.U.); 2 Fertility Centers of Illinois, Glenview, IL 60026, USA; hobeika.elie.md@gmail.com; 3 Department of Obstetrics and Gynecology, University of Chicago, 5841 South Maryland Ave, Chicago, IL 60637, USA

**Keywords:** infertility, mesenchymal stem cells (MSCs), reproductive system, stem-cell therapy

## Abstract

Female infertility is a global medical condition that can be caused by various disorders of the reproductive system, including premature ovarian failure (POF), polycystic ovary syndrome (PCOS), endometriosis, Asherman syndrome, and preeclampsia. It affects the quality of life of both patients and couples. Mesenchymal stem cells (MSCs) have received increasing attention as a potential cell-based therapy, with several advantages over other cell sources, including greater abundance, fewer ethical considerations, and high capacity for self-renewal and differentiation. Clinical researchers have examined the therapeutic use of MSCs in female infertility. In this review, we discuss recent studies on the use of MSCs in various reproductive disorders that lead to infertility. We also describe the role of microRNAs (miRNAs) and exosomal miRNAs in controlling MSC gene expression and driving MSC therapeutic outcomes. The clinical application of MSCs holds great promise for the treatment of infertility or ovarian insufficiency, and to improve reproductive health for a significant number of women worldwide.

## 1. Introduction

Female infertility is defined as the inability to achieve any pregnancy after one year (or longer) (Hull, Glazener et al., 1985) [1]. Infertility affects millions of people worldwide and thus has received intense attention, with clinical/ researchers focused on developing new therapies to prevent and treat infertility and improve quality of life of patients and their partners [2]. Various factors can cause female infertility, including several reproductive system disorders that impair the function of reproductive organs. The ovary is an intricate, highly regulated reproductive organ, that has both gametogenic and secretory functions. Through folliculogenesis, the ovary produces mature oocytes enclosed within fluid-filled follicles that produce and respond to various hormones and growth factors. Ovarian function is dependent on an array of coordinated autocrine, endocrine, and paracrine signaling pathways. Ovarian dysfunction can result from a number of reproductive system disorders, which lead to not only infertility, but also systemic health complications [3,4,5]. 

Several laboratory studies and clinical trials are investigating stem cells as a strategy for treating ovarian dysfunction and endometrial disorders that lead to infertility. In particular, several studies have focused on mesenchymal stem cells (MSCs) as an experimental approach to restoring ovarian function and treating infertility [6,7,8,9]. There are various sources of MSCs, such as bone marrow, fat tissue, amniotic fluid, umbilical cord tissue, placental tissue, menstrual blood, salivary gland, Wharton jelly, dental pulp and pluripotent stem cells [10,11]. The therapeutic potential of MSCs is due to its differentiation into multiple cell lineages and regulation of immune responses via immunomodulation [12] MSCs can differentiate into epithelial, stromal, and endothelial cells, and have been shown to restore endometrial function and improve pregnancy outcomes [13]. Several studies have reported that MSC transplantation improves ovarian function and ovarian reserve, and this action may be mediated by paracrine signaling pathways [14]. However, previous studies have also suggested that the number of differentiated MSCs is not sufficient to account for the observed improvement in fertility, and controversy remains regarding the differentiation of MSCs into oocytes after migrating to target tissue [5]. Here, we first review the various reproductive system disorders that cause infertility in women, then review the progress that has been made and the remaining challenges we face in applying MSCs to the treatment of these disorders. This information may help guide future laboratory experiments and clinical trials of MSCs as a promising therapy for restoring fertility, which affects a significant number of women all over the world.

## 2. Female Reproductive System Disorders

**Figure 1:**
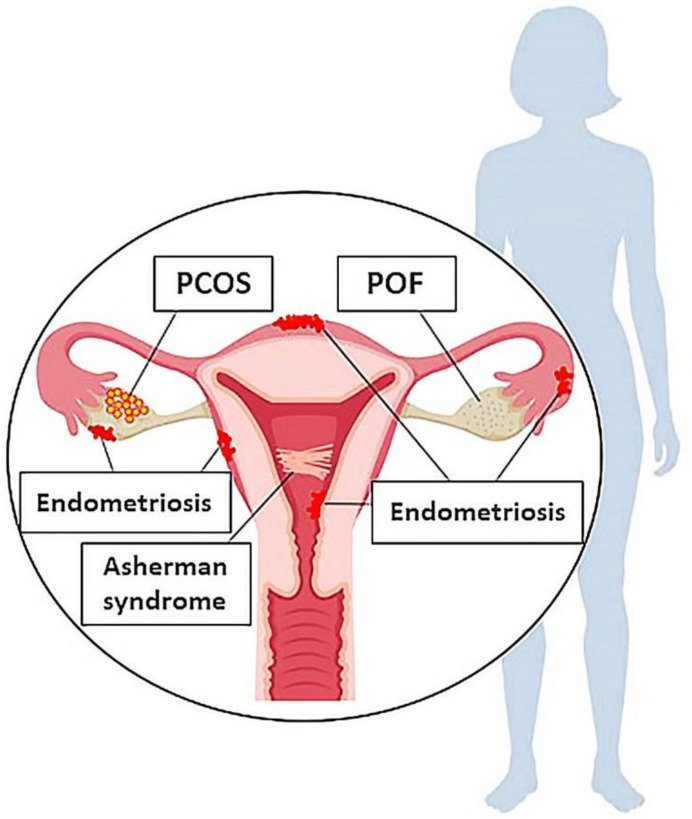
A schematic representation of the most important diseases of female reproductive system (including POF, PCOS, endometriosis, and Asherman syndrome). POF, Premature Ovarian Failure; PCOS, Polycystic Ovary Syndrome.

Disorders of the female reproductive system are associated with abnormalities in one or more the reproductive organs: ovaries, uterus, fallopian tubes, and cervix. These disorders can cause severe symptoms, including pain, frequent urination, altered menstruation, and are linked to negative reproductive outcomes, such as miscarriage and infertility. In this section, we briefly review five of the most common disorders that lead to infertility in women: premature ovarian failure (POF), polycystic ovary syndrome (PCOS), endometriosis, Asherman syndrome, and preeclampsia [3,4,5] (Figure 1).

### 2.1. POF

POF, also called primary ovarian insufficiency, presents with amenorrhea, hypergonadotropism, and estrogen deficiency, followed by infertility, prior to the age of 40 years. It is estimated that POF affects 1% of all women worldwide [15]; however, its incidence has increased in recent years [16]. POF is a heterogeneous disorder due to its multicausal pathogenesis, involving genetic, infectious, enzymatic, and iatrogenic factors. Some POF patients have idiopathic POF, with no identified etiology [17]. Available therapies for POF have low efficacy and may cause significant side effects, and there is a clear need for more comprehensive therapy to restore ovarian function in those with POF [16].

### 2.2. PCOS

PCOS is a common endocrine condition characterized by excess ovarian function and chronic anovulation, which can affect female fertility. PCOS is usually characterized by high androgen levels, altered menstruation, and small cysts on one or both ovaries. It is estimated that at least 7% of adult women struggle with this disorder. Remarkably, 5% to 10% of women 18-44 years of age have PCOS, making it the most common endocrine disorder among women of reproductive age. Recent investigations have shown that PCOS is linked to low-grade chronic inflammation [18,19]. Therefore, approaches to modulate the immune system and inflammatory pathways may be useful in treating PCOS.

### 2.3. Endometriosis

The endometrium is a thick, blood vessel-rich layer of tissue that develops on the surface of the uterus during the menstrual or estrous cycle. It is the deepest lining of the uterus, acts to preserve the uterine cavity patency, and is essential for early embryo implantation and growth. It is sloughed off and regenerated in every reproductive cycle. It contains a basal and a functional zone that are regulated by estrogen and progesterone [20,21]. Endometriosis is a chronic gynecological disorder characterized by the presence of endometrium tissue growing outside of the uterus. Ectopic endometrial tissue can be found on the ovaries, the uterosacral and broad ligaments, and the pelvic peritoneum. The typical outcomes of this disorder are pelvic pain and infertility. The prevalence of endometriosis is between 6% to 10%, and it is a multifactorial disorder [22,23], with a combination of genetic, environmental, and immune factors determining the risk of endometriosis. Several studies have suggested that the development of endometriosis is possibly a polygenetic process driven by parallel modifications in many biological pathways that allow endometrial cells to attach to the pelvic peritoneum, proliferate, and invade the underlying tissue [22,24].

### 2.4. Asherman syndrome

Asherman syndrome is an acquired condition identified by intrauterine adhesions, hypomenorrhea, and infertility. Scar tissue or adhesions form inside the uterus and impair implantation of the blastocyst, leading to recurrent miscarriage or infertility [25]. Asherman syndrome is often undetectable by routine tests or examinations. It is estimated that more than 90% of women with Asherman syndrome develop it after pregnancy-related curettage. Indeed, its prevalence is estimated to be up to 13% in women who underwent an abortion and 30% in women who underwent dilation and curettage after a late spontaneous abortion. Although this disease has been extensively studied, no effective treatments are available [25,26]. 

### 2.5. Preeclampsia

Preeclampsia is a complication of pregnancy that is increasing in prevalence around the world. It is identified by high blood pressure, proteinuria, and edema. Preeclampsia frequently develops after 20 weeks of pregnancy in females who had normal blood pressure. Its incidence is 3-7% among nulliparous women and 1-3% among multiparous women [27]. Preeclampsia is one of the main causes of preterm birth and fetal mortality and morbidity, and in low-income and middle-income countries, it is linked to 10-15% of fetal deaths. While preeclampsia has a complex pathophysiology, most clinical studies propose that the placenta plays a central role [28,29]. There are applicable guidelines for clinicians treating preeclampsia to reduce maternal and neonatal morbidity [29].

## 3. Mesenchymal Stem cells (MSCs)

MSCs have received increasing attention for their potential therapeutic utility in various female reproductive system disorders. The Mesenchymal and Tissue Stem Cell Committee of the International Society for Cellular Therapy introduced specific criteria for MSCs used in research and for clinical applications [30]. First, MSCs should be plastic-adherent when preserved in standard cell culture media. Second, MSCs should have a specific gene expression pattern with expression of the surface molecules CD73, CD90, and CD105, and without the expression of CD11b, CD14, CD19, CD34, CD45, and CD79a. Finally, MSCs should have the ability to differentiate into various cell types, including adipocytes, osteoblasts, and chondroblasts, in cell culture conditions [30,31,32]. The various MSCs are categorized according to their source.

Several studies have examined the application of MSCs for treating fertility, in particular, by restoring ovarian function in the context of various gynecologic disorders [6,7,8,9]. MSCs have been shown to spontaneously translocate to the injured ovary and attach and proliferate in response to various growth factors and hormones. These studies have reported that MSCs improve ovarian function and help ovarian functional recovery, but it remains unclear whether this effect is achieved through differentiation of MSCs into oocytes or supporting follicular or stromal cells after migration to the ovary [5].

Many studies indicated that the reproductive treatment effect of BMSC is linked to their secretome, which is rich in bioactive factors that support in ovarian function [33]. These molecules may include insulin-like growth factor (IGF), vascular endothelial growth factor (VEGF), and other growth factors that induce cell growth, differentiation, and immunoregulation to restore ovarian function [34]. Some data further suggest that the paracrine activity of MSCs has a more significant effect on ovary function than its stimulatory effects on cell growth and differentiation [5,34]. Growing evidence in animal studies and preliminary clinical trials has revealed that MSCs also have immunomodulatory effects through interaction with immune cells. Importantly, MSC paracrine function is tightly regulated by RAP1/NFkb signaling pathway [35]. NFkb signaling pathway is highly active in reproductive system disorders accompanied with inflammation. These effects seem to be related to pathways that induce regeneration and angiogenesis, which may also improve ovarian function. This potential mechanism of MSCs activity is of particular importance, since these pathways can regulate immune responses and inflammation, improve injured tissues, and stimulate progenitor cells to differentiate into specific tissue cells [5,36].

Additionally, miRNAs and exosome transfer show another novel mechanism by which MSCs exert functions through transfer of mitochondria, a key player involved in many biological processes in health and disease, including in POF. Some pro-inflammatory cytokines, i.e. IL-6 and TNF-α, can induce MSC skeletal re-arrangement and form tunneling nanotubes (TNT) through which mitochondria mobility occurs from MSC to neighbor cells, such as airway epithelia cells, cardiomyocytes, and retinal ganglion cells. Inflammation-driven mitochondrial transfer of MSC to reproductive system cells including oocytes were also reported recently [37]. The therapeutic effects of MSC and direction of mitochondrial transfer highly depend on a niche where MSC is located. It appears that a pro-inflammatory environment can enhance MSC mitochondrial transfer into T cells, in turn, can educate immune cells to control the inflammatory response [38]. Here, we review studies of the biology and clinical utility of MSCs from several different sources to treat female reproductive system disorders.

### 3.1. Bone Marrow Stromal Cells (BMSCs)

BMSCs comprise a heterogeneous collection of cells that support hematopoietic cells. This class of stem cells was first described by Owen and Friedenstein, who isolated them from nucleated bone marrow cells in 1988 [39,40]. BMSCs are an important source of multipotent stem cells and serve as a standard for comparison of MSCs from different sources [10]. 

Many studies have examined the functional nature and the differentiation capacity of BMSCs. These cells can differentiate into chondroblasts, osteoblasts, and adipocytes. Findings have shown that these cells account for only a limited number of nucleated bone marrow cells, due to their long replication cycle [10]. Interestingly, BMSCs also have the ability to differentiate into endometrial [41,42], endothelial [43], and granulosa cells (GCs) [44]. Under specific circumstances, these cells can also form new bone after bone marrow transplant. The finding that these cells can be manipulated in experimental investigations and consequently form bone in clinical applications offers an important model system for testing therapeutic strategies aimed at improving reproductive dysfunction [45]. 

To address the protective effects of BMSCs in improving ovarian function, it should be first noted that chemotherapy, despite its beneficial effects in cancer therapy, may lead to ovarian dysfunction and infertility. Using this model, findings have shown that BMSCs improve ovarian function and reduce ovarian failure [46,47,48,49]. Abd-Allah et al., reported that BMSCs improved ovarian function in cyclophosphamide-induced POF; BMSC treatment induced VEGF expression, increased estradiol levels, restored ovarian structure, and decreased expression of the apoptotic factor Caspase-3 [46]. Another investigation showed that BMSC treatment increased fertility and reduced ovarian dysfunction in mice after chemotherapy [49]. These remarkable effects of BMSCs may be due to angiogenic and growth factors secreted from these cells [15].

Other evaluations have reported that BMSCs can migrate to the uterus and induce the restoration and regeneration of damaged endometrium in human and animal models [41,50,51,52]. Indeed, Singh et al., showed the therapeutic effects of BMSC in the restoration of menstruation in a model of Asherman syndrome [53]. Other studies revealed that CD133+BMSCs can increase the proliferation rate of endometrial tissue by engrafting around blood vessels of the target cells, and producing IGF1, thrombospondin 1, and other growth factors [54,55]. Moreover, several findings demonstrated that BMSC replacement was capable of improving endometrium dysfunction and infertility in patients with Asherman syndrome, by increasing endometrial receptivity, inducing angiogenesis and suppressing fibrosis [55,56,57].

**Figure 2:**
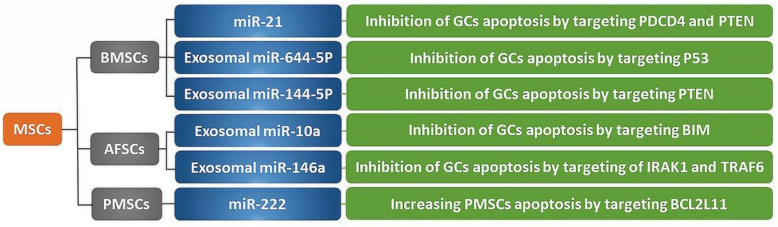
The association between miRNAs and different MSCs with respect to their effects on the female reproductive system. MSCs, Mesenchymal Stem Cells; BMSCs, Bone Marrow Stromal Cells; AFSCs, Amniotic Fluid Stem Cells; PMSCs, Placenta Mesenchymal Stem Cells; miR, miRNAs, microRNA; GCs, Granulosa Cells; PDCD4, Programmed Cell Death Protein 4; PTEN, Phosphatase and Tensin Homolog; BIM, Bcl-2-Like Protein 11; IRAK1, Interleukin-1 Receptor-Associated Kinase 1; TRAF6, TNF Receptor-Associated Factor 6; BCL2L11, B-Cell Lymphoma 2 Like 11.

Other lines of investigation have focused on the molecular and cellular mechanisms of action driving the observed effects of BMSCs in various disease models. A recent study showed that microRNAs (miRNAs) play a critical role in regulating stem cell differentiation and regeneration by inhibiting target mRNA translation [58]. These small non-coding RNAs may play a similar role in regulating the physiological and pathological mechanisms of stem cells and outcomes related to the ovarian function (Figure 2) [15]. Fu et al., found that overexpression of miR-21 in BMSCs is linked to follicular growth and apoptosis of GCs in female rats with cyclophosphamide-induced POF [48]. GCs are somatic steroidogenic cells that surround oocytes and are essential for their growth and development. GCs also support oocytes by producing nutrients and growth factors. GC death results in the abnormal development of oocytes [59]. The effect of miR-21 overexpression in BMSCs may be mediated by a reduction in the expression of programmed cell death protein 4 (PDCD4) and phosphatase and tensin homolog (PTEN). Moreover, miR-21 overexpression was associated with a higher level of estradiol and a lower level of follicle-stimulating hormone (FSH) [48]. Exosomes are a subset of membrane-bound extracellular vesicles that can be secreted by stem cells. They enclose various cellular compounds, including, proteins, lipids, and non-coding RNAs such as miRNAs. Exosomes are important vehicles for intercellular communication, and as such, are critical for many physiological functions. Recent studies indicated that exosomal miRNAs can regulate intercellular signal transduction and control molecular activities in different diseases [60,61,62,63]. Two studies have demonstrated a role of miRNAs carried by BMSCs-derived exosomes in promoting the recovery of ovarian function in a POF animal model [61,62]. Sun et al., reported that miR-644-5p carried by BMSCs-derived exosomes controls p53 signaling, thereby suppressing the death of GCs [61]. A more recent research described that BMSC-derived exosomal miR-144-5p is capable of restoring ovarian function by regulating PTEN after chemotherapy-induced POF in rats [62]. Together, these findings suggest that miRNA-regulated gene expression underlies BMSC-based therapy outcomes.

### 3.2. Adipose-Derived Stem Cells (ADSCs)

ADSCs are a newer source of MSCs, that have been applied successfully to tissue regeneration [64,65,66]. They also have some notable advantages over MSCs derived from other sources, such as BMSCs. For instance, they can be easily isolated by minimally invasive methods and have immunosuppressive functions. ADSCs have the same differentiation capacity as other MSCs, producing adipocytes, osteoblasts, and chondroblasts, as well as neural cells, cardiomyocytes, myocytes, vascular endothelial cells, and hepatocytes. Hence, these cells have the potential for broad applications in clinical settings [67,68,69].

Previous data have demonstrated that ADSC-based therapy upregulates VEGF expression, promotes new blood vessel growth, and improves ovarian graft quality in rats [70]. Moreover, ADSCs improved ovarian function in mice after chemotherapy, increasing the number of new blood vessels and ovarian follicles [71,72]. Adding a collagen scaffold increased the short-term preservation of ADSCs in the ovaries of female rats with POF compared to ADSC therapy alone [73]. In addition, ADSCs were found to increase the number of follicles, improve ovulation, and reduce GCs death in cyclophosphamide-induced POF [15]. Importantly, another study isolated allogeneic ADSCs from women with endometriosis to investigate their effects on endometriosis-derived cells in vitro. However, they concluded that allogeneic ADSCs should not be used as a therapeutic strategy for patients with endometriosis, since they may induce the growth of ectopic endometrial tissue and support the development of endometriosis [74]. Interestingly, using ADSCs along with estrogen increased endometrial regeneration in a female rat model of Asherman syndrome [75]. 

These findings suggest that ADSCs are one of the most important therapeutic cells for recovery of ovarian function, though technical challenges in replacement therapy still need to be addressed. 

### 3.3. Menstrual Blood-Derived Mesenchymal Stem Cells (MenSCs)

MenSCs are a new source of MSCs that have attracted significant attention since their discovery in 2007. MenSCs are collected from menstrual blood and show some exclusive characteristics of better-known stem cells and offer an alternative and plentiful source of cells for clinical applications in tissue regeneration. MenSCs are easy to harvest using noninvasive techniques and have a high proliferation rate. There have been no reports of autoimmune rejection responses to MenSCs [76,77]. Several investigations have shown beneficial effects of MenSCs in different diseases, including neurodegenerative disorders [77,78,79]. Remarkably, some studies have reported a protective role of MenSCs against GCs death in the ovarian interstitium of an animal model of POF. In these studies, MenSCs restored ovarian function [80,81]. It was suggested that this effect is likely mediated by a decrease in the expression of growth arrest and DNA damage inducible beta (GADD45B), a stress sensor involved in cell cycle control, and by an increase in the expression of cell division control 2 (CDC2) and cyclin B1 as cell cycle inducers [82,83,84,85]. Moreover, another study showed that MenSCs treatment led to higher levels of fibroblast growth factor 2 (FGF2), with beneficial effects on ovarian structure and function [86].

The endometrium comprises a subset of cells presenting MenSCs properties. Interestingly, MenSCs collected from ectopic endometriotic lesions appear to promote the pathogenesis of endometriosis [87,88]. Despite this, various studies have reported on the use of MenSCs to improve endometrial growth and proliferation in the injured endometrium and reducing inflammation and fibrosis through Wnt family member 5a (Wnt5a) and growth differentiation factor 5 (Gdf5) growth factors and the Hippo signaling pathway [89]. For instance, in 2018, Zheng et al., were the first to demonstrate the ability of MenSCs to differentiate into endometrial cells in the laboratory and rebuild endometrial tissue in animal models in response to estrogen and progesterone [90]. Moreover, MenSCs treatment, along with hormonal stimulation, was shown to improve endometrium structure and fertility potential in women with Asherman syndrome [91]. Domnina et al., reported that MenSCs treatment increases fertility through induction of angiogenic and anti-inflammatory factors in a rat model of Asherman syndrome [92]. Other work has found that MenSCs produce functional spheroids, which appear to be essential for their observed therapeutic effects [93]. 

### 3.4. Umbilical Cord Mesenchymal Stem Cells (UC-MSCs)

UC-MSCs are a rich source of MSCs that express stem cell-specific markers and can be differentiated into several mesodermal cell types for tissue repair and regulation of the immune response. US-MSCs have some notable features, including fast self-renewal, lower oncogenicity, poor immunogenic properties, and ability to collect them in a painless and non-invasive fashion [94]. Their poor immunogenic properties are due to a lower expression of major histocompatibility complex (MHC) class I and class II proteins needed for adaptive immunity. Considering these features, UC-MSCs have emerged as a superior source of stem cells for transplantation compared to other sources of MSCs. Several methods have been developed for isolating these cells from Wharton’s jelly, arteries, or veins [15,94,95,96]. 

Some groups have reported that UC-MSCs can activate primordial follicles, increase ovarian function, and decrease ovarian cell death in both animal and human models of POF, particularly in the presence of collagen [97,98,99,100]. After the transplantation of CM-Dil-labeled UC-MSCs into the ovaries of mice with cyclophosphamide-induced POF, the cells were unequally distributed within the ovary, with a greater number of cells located in the medulla rather than in the cortex and germinal epithelium [101]. 

Several factors and pathways may underlie the protective effects of UC-MSCs. For instance, some studies suggest that UC-MSCs likely decrease GCs apoptosis by affecting the Mitogen-Activated Protein Kinase (MAPK) signaling pathway, G-protein coupled receptor (GPCR) signaling pathway, and insulin signaling pathway [15]. In 2016, Elfayomy et al., showed that UC-MSCs reduced ovarian cell death by regulating critical molecules involved in inhibiting Caspase-3‒induced apoptosis [102]. Other research found that UC-MSCs on a collagen scaffold activated primordial follicles by phosphorylation of the transcription factors Forkhead box protein O1 (FOXO1) and FOXO3a [103,104]. Furthermore, UC-MSCs have been reported to induce angiogenic growth factors, including VEGF, hepatocyte growth factor (HGF), placental growth factor (PGF), and transforming growth factor-beta 1 (TGF-β1) [15,105].

New evidence suggests that the potential utility of UC-MSC transplantation improves pathological alterations in PCOS. Researchers found that UC-MSC transplantation restored ovarian function in dehydroepiandrosterone (DHEA)-induced PCOS in mice. This effect was mediated by the downregulation of inflammatory cytokine expression, including interleukin 1 beta (IL-1β), tumor necrosis factor alpha (TNF-α), and interferon gamma (IFN-γ), as well as fibrosis-related genes such as connective tissue growth factor (CTGF) [36]. While these studies suggest that UC-MSC transplantation may be helpful for controlling disorders related to the ovaries, more studies are needed to develop a valid US-MSC‒based therapeutic approach.

Several studies have investigated the effects of UC-MSCs in endometrial regeneration. UC-MSCs were found to restore injured endometrial tissue in cesarean delivery scars [106,107], and remarkably, the differentiation of UC-MSCs into endometrial cells was observed in a recent study [108]. It has been demonstrated that UC-MSCs improved endometrial injury and infertility by inhibiting inflammation and excessive fibrosis, and increasing cell proliferation and vascular marker expression [109]. Recent findings showed that UC-MSCs on a collagen scaffold restored the endometrium by inducing the expression of matrix metalloproteinase 9 (MMP9) in rats [110,111]. Furthermore, UC-MSCs on a collagen scaffold were useful in treating patients with recurrent uterine adhesions through the induction of von Willebrand factor (VWF), Ki67, vimentin, and estrogen receptor alpha (ERα) gene expression, thereby increasing cell proliferation and differentiation [112].

Remarkably, several studies have demonstrated positive effects of UC-MSC transplantation on preeclampsia. In a lipopolysaccharide (LPS)-induced preeclampsia rat model, UC-MSC transplantation improved preeclampsia symptoms and reduced the levels of inflammatory cytokines such as TNF-α and IL-6 [113]. This effect of UC-MSCs was also observed in an endotoxin-induced preeclampsia rat model, with a decrease in TNF-α and IL-1β levels [114]. Moreover, UC-MSC improved angiotensin receptor agonistic autoantibody (AT1-AA)-induced pregnancy hypertension and reduced the serum levels of TNF-α, providing further evidence that UC-MSCs may be a possible therapy for preeclampsia [115]. In another study, trophoblast cells were treated with UC-MSC to investigate the effects on migration and proliferation. The investigators observed a protective effect of UC-MSC on trophoblast cellular functions, opening a new avenue for treating placenta-related disorders like preeclampsia [116]. Interestingly, exosomes derived from UC-MSC improved the morphology and angiogenesis of the placenta in a dose-dependent manner in a rat model of preeclampsia [117]. Taken together, UC-MSCs may be beneficial for preeclampsia therapy, and further studies are warranted.

### 3.5. Amniotic Fluid Stem Cells (AFSCs)

Amniotic fluid supports fetal growth and development by providing essential nutrients during embryogenesis and gestation [46]. Amniotic fluid is also a novel source of stem cells with potentially broad applications. AFSCs are not only appropriate for use as therapy, but may have clinical value for the diagnosis of ovarian dysfunction. These cells lack the ethical limitations of embryonic stem cells and show immunomodulatory characteristics. AFSCs can be collected easily and be differentiated into several cell types, including bone, muscle, and adipose cells for use in regenerative treatments [118]. The capacity of AFSCs to repair bone, cartilage, and muscle has been investigated in several animal models [119,120,121,122].

Previous data have indicated that AFSCs may modulate ovarian function by regulating paracrine signaling pathways, such as TGF-α, TGF-β, FGF1, VEGF, bone morphogenetic protein 4 (BMP-4), and epidermal growth factor (EGF) pathways [15]. Thus, although AFSCs do not differentiate into GCs in vivo, AFSCs have been shown to improve ovarian function in a mouse model of POF by suppressing follicular atresia and preserving healthy follicles [123]. Moreover, Liu et al., reported that a subpopulation of CD4C/CD105+ AFSCs improved the regeneration of ovarian cells after chemotherapy-induced POF in mice [124]. 

Considering the role of exosomal miRNAs in the restorative effects of MSCs in the ovaries, the potential role of exosomal miR-10a and miR-146a derived from AFSCs has been investigated. In this report, two AFSC exosomal miRNAs increased the survival of GCs and improved follicular atresia by targeting Bcl-2-like protein 11 (BIM), interleukin-1 receptor-associated kinase 1 (IRAK1), and TNF receptor-associated factor 6 (TRAF6), after chemotherapy-induced POF. Moreover, the downregulation of miR-10a and miR-146a suppressed their protective effects on the survival of injured GCs. Importantly, the administration of miR-10a and miR-146a restored the effect on GCs survival, with miR-10a showing a greater effect. These data suggest that these and possibly other miRNAs may offer a new therapeutic strategy for modulating and treating ovarian dysfunction [125]. However, there are some limitations to their clinical utility. The systemic administration of miRNAs in high doses may have some dangerous side effects, based on their known roles in various physiological and pathophysiological mechanisms. To address this challenge, investigators found that the local injection of miRNAs into areas of tissue damage could elicit benefits while reducing side effects, as these small molecules have a short half-life [125,126].

### 3.6. Amnion-Derived Mesenchymal Stem Cells (AD-MSCs)

AD-MSCs are another potential cell source for regenerative medicine and transplantation. They can be easily collected from human amnion using noninvasive methods. Two recent studies demonstrated their beneficial effects on ovarian function in a chemotherapy induced POF rat model [34,127]. They reported that AD-MSCs can protect against chemotherapy-induced POF by decreasing GCs apoptosis and increasing cell proliferation and angiogenesis through paracrine signaling pathways in the ovarian microenvironment. Furthermore, AD-MSCs inhibited chemotherapy-induced inflammation by reducing inflammatory cytokines, such as IL-1β, IL-6, and TNF-α in the ovaries. Indeed, in a study using low-intensity pulsed ultrasound (LIPUS)-pretreated AD-MSCs, the expression of several growth factors, including IGF1, FGF2, and VEGF, reduced the expression of IL-1β, IL-6, and TNF-α in the ovaries of POF rats. Interestingly, LIPUS pretreatment with AD-MSC transplantation was more effective in decreasing chemotherapy-induced inflammation and apoptosis in the ovarian tissue of POF rats [128]. However, Hwang et al., examined the levels of monocyte chemotactic protein-1 (MCP-1), one of the key chemokines in normal and preeclamptic pregnancies but observed no significant difference in its expression in AD-MSCs [129]. Collectively, AD-MSC transplantation appears to reduce ovarian injury and may be a novel therapy for disorders such as POF to improve reproductive health.

### 3.7. Placenta Mesenchymal Stem Cells (PMSCs) 

PMSCs are a new class of stem cells for transplantation, with the potential to restore ovarian function in POF. They can be isolated easily and have poor immunogenic properties. They also have some remarkable advantages over other MSCs, due to their high differentiation and proliferation potential, making them an attractive cell source for transplantation and regenerative medicine [15]. This new class of stem cells was shown to improve ovarian function in POF mice via the regulation of cytokines. Moreover, PMSCs were able to reduce the levels of estradiol, FSH, and luteinizing hormone (LH) and induce the expression of FSH receptor (FSHR) and anti-Müllerian hormone (AMH) in POF mice. FSHR mediates FSH signaling during folliculogenesis. AMH also plays a key role in the regulation of folliculogenesis and is an indicator of the ovarian reserve. The same study also revealed that the PI3K/Akt signaling pathway contributes to the effects of PMSC transplantation on ovarian function [130,131,132]. In addition, Li et al., reported that PMSC transplantation decreased GCs apoptosis through the inhibition of the endoplasmic reticulum (ER) stress inositol-requiring enzyme 1 (IRE1) signaling pathway, thereby improving ovarian structure and function in POF mice [133]. Furthermore, PMSC transplantation was shown to improve ovarian function in ovariectomized rats by inducing the production of estrogen and the expression of folliculogenesis-related genes [134].

Stem cell transplantation has great potential for the treatment of preeclampsia as a pregnancy-specific hypertensive disorder. PMSCs produce a wide array of inflammatory cytokines and growth factors that play key roles in modulating the signaling pathways underlying preeclampsia [129,135,136,137,138]. Moreover, previous findings have demonstrated that miRNAs such as miR-222 influence PMSCs differentiation and activity in preeclampsia, by targeting B-cell lymphoma 2 like 11 (BCL2L11) [139]. It was also reported that PMSCs interfere with the regulators of the G1/S phase cell cycle checkpoint in patients with preeclampsia [140]. Therefore, PMSCs may provide a future therapeutic tool for placenta-related diseases like preeclampsia. Nevertheless, more studies are needed to decipher their exact role in the etiopathology of the disorder.

### 3.8. Pluripotent Stem Cell-derived MSCs (PSC-MSCs)

MSCs can also be derived from pluripotent stem cells (PSCs), which have capabilities of rejuvenation of tissues [11]. In 2010, Lian et al., showed that PSC-MSCs can be differentiated into osteoblasts, adipocytes, chondrocytes and promote angiogenesis [141]. The literature also reported that MSCs derived from PSCs have better survival rates after engraftment in disease condition. It is due to higher telomerase activity of PSC-MSCs with less senescence property compared to the BM-MSCs. MSCs derived from pluripotent stem cells have higher proliferative potential and showed stronger immunomodulation properties than bone-marrow-derived MSCs [142]. PSC-MSCs are devoid of teratoma formation inherent to its parental cell population, even with their higher proliferation than BM-MSCs [143]. Recent studies using GMP-grade MSCs derived from iPSCs have been used in refractory graft-versus-host-disease (GVHD) in clinical trials [144], though many female reproductive system disorders are related to aberrant inflammation and abnormal immune states. Therefore, PSC-MSCs may provide another putative cellular source for infertility disorders, due to strong immunomodulatory properties [142].

From this study we can summarize the effect of different MSCs in female reproductive diseases (Table 1).

## 4. Conclusions 

MSCs have shown great potential for treating female infertility in various animal models and clinical studies. MSCs exert their effects through the modulation of various molecular and biological pathways. miRNAs and exosomal miRNAs in particular appear to play an important role in mediating MSCs effects and are thus novel therapeutic targets for further study. Studies have advanced our understanding of the mechanisms and therapeutic potential of stem cell-based therapies for gynecologic disorders that alter reproductive tissue function. These studies have also opened opportunities for the development of novel and effective MSC-based treatments, with the potential to help women with infertility or ovarian insufficiency by restoring their reproductive health and improving their quality of life. The utilization of MSC in female infertility treatment is at stages of pre-clinical research or a very early clinical trial phase. The safety and efficacy of MSC in infertility treatment require further investigation. Different disease conditions between the animal model and human, MSC quality and dosage and routes of MSC delivery must be carefully evaluated. Further studies of MSCs function in transplantation and regenerative medicine are needed. 

## Figures and Tables

**Table 1:** Effects of different MSCs on female reproductive diseases.

MSC Types	Disease	Model	Main Effect	References
**BMSC**	POF	Rabbits	Increasing the secretion of VEGF	[46]
	POF	Mice	Formation of new primordial follicles	[49]
	POF	Rat	Increasing ovarian weight, follicle counts and E_2_ levels	[48]
	POF	Rat	Inhibition of GCs apoptosis	[61]
	POF	Rat	Increasing follicle counts, E_2_ and AMH levels	[62]
	Asherman syndrome	Human	Restoration of menstruation in endometrium	[53]
	Asherman syndrome	Rat	Reconstruction of functional endometrium	[41]
	Asherman syndrome	Murine	Regeneration of endometrium	[56]
	Asherman syndrome	Human	Reconstruction of functional endometrium	[57]
**ADSC**	POF	Mice	Increasing the number of follicles with normal structure	[71]
	POF	Mice	Increasing follicles at different stages and ovulation	[72]
	POF	Rat	Increasing follicle counts, E_2_ levels and pregnancy rates	[73]
	Endometriosis	Cell culture	Supporting the development of endometriosis	[74]
	Asherman syndrome	Rat	Regeneration of endometrium	[66]
**MenSC**	POF	Mice	Increasing ovarian weight, follicle counts and E_2_ levels	[80]
	POF	Rat	Increasing AMH, E_2_ and progesterone levels	[81]
	POF	Mice	Inhibition of GCs apoptosis	[77]
	Endometriosis	Mice	Increasing the invasion and angiogenesis	[86]
	Asherman syndrome	Human	Regeneration of endometrium	[91]
	Asherman syndrome	Rat	Increasing the secretion of angiogenic and anti-inflammatory factors	[92]
**UC-MSC**	POF	Murine	Increasing ovarian weight, follicle counts, AMH levels, expression of Inhibin A and FSHR in growing follicles	[97]
	POF	Rat	Inhibition of GCs apoptosis, improvement of endocrine secretion system and folliculogenesis,	[98]
	POF	Rat	Recovery of estrous cycle, levels of sex hormones and fertility	[99]
	POF	Mice	Inhibition of GCs apoptosis, increasing the level of sex hormons and number of follicles	[100]
	POF	Human	Activation of primordial follicles	[103]
	POF	Mice	Increasing ovarian volume and angiogenesis, number of antral follicles, AMH and E_2_ levels	[104]
	POF	Rat	Increasing follicle counts and E_2_ levels	[102]
	PCOS	Mice	Inhibition of ovarian local and systemic inflammatory responses	[36]
	Preeclampsia	Rat	Inhibition of inflammation	[113]
	Preeclampsia	Rat	Inhibition of inflammation	[114]
	Preeclampsia	Rat	Inhibition of hypertension and inflammation	[115]
	Preeclampsia	Rat	Improvement of morphology and angiogenesis of placenta	[117]
**AFSC**	POF	Mice	Inhibition of follicular atresia and preserving the healthy follicles	[123]
	POF	Mice	Regeneration of ovarian cells	[124]
	POF	Mice	Inhibition of GCs apoptosis and follicular atresia	[125]
**AD-MSC**	POF	Rat	Inhibition of GCs apoptosis, increasing ovarian angiogenesis and follicular development	[34]
	POF	Rat	Inhibition of GCs apoptosis, increasing follicular numbers and AMH levels	[127]
	POF	Rat	Inhibition of inflammation	[128]
**PMSC**	POF	Mice	Inhibition of GCs apoptosis, increasing E_2_ levels	[130]
	POF	Mice	Inhibition of GCs apoptosis, improvement of ovarian function	[131]
	POF	Mice	Increasing follicular numbers, E_2_ and AMH levels	[132]
	POF	Mice	Inhibition of GCs apoptosis	[133]
	Preeclampsia	Human	Increasing the production of HGF	[137]

MSC, Mesenchymal Stem Cell; BMSC, Bone Marrow Stromal Cell; ADSC, Adipose-Derived Stem Cell; MenSC, Menstrual Blood-Derived Mesenchymal Stem Cell; UC-MSC, Umbilical Cord Mesenchymal Stem Cell; AFSC, Amniotic Fluid Stem Cell; AD-MSC, Amnion-Derived Mesenchymal Stem Cell; PMSC, Placenta Mesenchymal Stem Cell; POF, Premature Ovarian Failure; PCOS, Polycystic Ovary Syndrome; GC, Granulosa Cell; E_2_, Estradiol; AMH, Anti-mullerian hormone.
